# Canonical Wnt signaling induces BMP-4 to specify slow myofibrogenesis of fetal myoblasts

**DOI:** 10.1186/2044-5040-3-5

**Published:** 2013-03-05

**Authors:** Kazuki Kuroda, Shihuan Kuang, Makoto M Taketo, Michael A Rudnicki

**Affiliations:** 1 Sprott Center for Stem Cell Research, Ottawa Hospital Research Institute, 501 Smyth Road, Ottawa, ON, K1H 8L6, Canada; 2 Present Address: Department of Animal Sciences, Purdue University, 174B Smith Hall, 901 W State St, West Lafayette, IN, 47907, USA; 3 Department of Pharmacology, Kyoto University Graduate School of Medicine, Konoe, Yoshida, Sakyo, Kyoto, 606-8501, Japan; 4 Present address: Division of Cell Biology and Neuroscience, Department of Morphological and Physiological Sciences, Faculty of Medical Sciences, University of Fukui, 23-3, Matsuoka-Shimoaizuki, Eiheiji, Fukui, 910-1193, Japan

**Keywords:** Skeletal muscle, Fetal myoblasts, Canonical Wnt signaling, BMP4 signaling, Differentiation, Slow muscle specification

## Abstract

**Background:**

The Wnts are secreted proteins that play important roles in skeletal myogenesis, muscle fiber type diversification, neuromuscular junction formation and muscle stem cell function. How Wnt proteins orchestrate such diverse activities remains poorly understood. Canonical Wnt signaling stabilizes β-catenin, which subsequently translocate to the nucleus to activate the transcription of TCF/LEF family genes.

**Methods:**

We employed TCF-reporter mice and performed analysis of embryos and of muscle groups. We further isolated fetal myoblasts and performed cell and molecular analyses.

**Results:**

We found that canonical Wnt signaling is strongly activated during fetal myogenesis and weakly activated in adult muscles limited to the slow myofibers. Muscle-specific transgenic expression of a stabilized β-catenin protein led to increased oxidative myofibers and reduced muscle mass, suggesting that canonical Wnt signaling promotes slow fiber types and inhibits myogenesis. By TCF-luciferase reporter assay, we identified Wnt-1 and Wnt-3a as potent activators of canonical Wnt signaling in myogenic progenitors. Consistent with *in vivo* data, constitutive overexpression of Wnt-1 or Wnt-3a inhibited the proliferation of both C2C12 and primary myoblasts. Surprisingly, Wnt-1 and Wnt-3a overexpression up-regulated BMP-4, and inhibition of BMP-4 by shRNA or recombinant Noggin protein rescued the myogenic inhibitory effect of Wnt-1 and Wnt-3a. Importantly, Wnt-3a or BMP-4 recombinant proteins promoted slow myosin heavy chain expression during myogenic differentiation of fetal myoblasts.

**Conclusions:**

These results demonstrate a novel interaction between canonical Wnt and BMP signaling that induces myogenic differentiation towards slow muscle phenotype.

## Background

Skeletal muscles of the trunk and limb, except for some craniofacial and esophageal muscles, are derived from somites during embryonic development [1-3]. Specification of somitic cells into myogenic lineages is regulated by positive and negative signals from the surrounding tissues. Wnt signaling induced by Wnt-1, -3a, -4, -6, -7a and 11 from dorsal neural tube or ectoderm is critical for the induction, initiation and progression of myogenesis in the presomitic mesoderm and early somites (Reviewed in [4,5]). Within the embryonic myogenic progenitors, Wnt also regulate the expression of Pax3/7, MyoD and Myf5, key transcription factors involved in myogenesis [6-10]. Importantly, genetic knockout studies have clearly demonstrated the requirement of several Wnt molecules and β-catenin in the normal development of skeletal muscles [11,12]. These diverse functions of Wnt are mediated by both a canonical signaling pathway that requires stabilization and nuclear translocation of β-catenin, and non-canonical pathway that is independent of β-catenin [5]. Therefore, canonical and non-canonical Wnt signaling pathways play multiple essential roles in embryonic myogenesis.

Wnt signaling is also involved in the regulation of postnatal satellite cell function and skeletal muscle regeneration. Satellite cells are muscle resident stem cells responsible for postnatal regeneration of injured muscles. During muscle regeneration, Wnt-5a and Wnt-7a induce muscle resident CD45^+^ stem cells to undergo myogenic specification and differentiation [13]. Wnt-7a also acts through the non-canonical Wnt signaling pathway to stimulate the symmetric expansion of satellite cells and promote skeletal muscle hypertrophy [14,15]. Similarly, canonical Wnt activation was shown to induce satellite cell proliferation during skeletal muscle regeneration [16]. However, contradictory results showing that activation of canonical Wnt signaling is necessary to counteract Notch signaling to induce myogenic differentiation were also reported [17]. Furthermore, in the aged niche, elevated systemic Wnt molecules impede myogenic differentiation and facilitate satellite cell fate conversion to fibroblastic cell lineages [18].

Adult skeletal muscles contain heterogeneous types of muscle fibers that can be broadly divided into slow- and fast-twitch myofibers [19]. In the limb muscles, the slow-twitch myofibers express type I myosin heavy chain (MyHC), while the fast-twitch myofibers express type IIa, IIx and IIb MyHC isoforms [19]. During early stages of myofiber generation in chicks, Wnt-11 promotes fast myofiber formation, whereas Wnt-5 enhances slow myofiber generation [20]. Wnt-4 similarly stimulated fast myofiber formation in chicks [21]. Activation of β-catenin induced myofiber hypertrophy followed by degeneration of fast myofibers in zebrafish [22]. In mice, depletion of β-catenin during myogenesis in Pax7-lineage cells led to reduced slow myofibers and overall reduction of muscle mass [23]. Interestingly, expression of stabilized β-catenin in Pax7 lineage cells also led to reduced myogenesis but increased slow myofibers [23]. These studies suggest that Wnt signaling plays diverse roles in regulating slow- versus fast-twitch myofiber formation during development. However, embryonic lethality of β-catenin deficient mice or over-expressing mice precludes analysis of Wnt signaling in postnatal muscles. In addition, how canonical Wnt signaling regulates myofiber types has remained unclear.

Myofibers in postnatal skeletal muscles retain an adaptive capacity to switch between slow- and fast-twitch properties that largely depend on motoneuron activity [19]. Wnt signaling also regulates the establishment and maintenance of neuromuscular junctions that connect motor neurons and myofibers. In Drosophila and mice, Wnt secreted by presynaptic motoneurons interact with Agrin-MusK to induce assembly of postsynaptic endplates (Reviewed in [24]). There is evidence that neuromuscular junctions are phenotypically and functionally distinct in fast and slow muscles [25,26]. Whether Wnt signaling is differentially activated in slow and fast myofibers is completely unknown.

In this study, we used transgenic Wnt-reporter mice and mice with constitutively activated canonical Wnt signaling specifically within skeletal muscle to investigate the function of canonical Wnt signaling in muscle. We found that Wnt signaling is highly activated in prenatal muscle and rapidly declines in postnatal muscle, with some residual activation limited to the slow myofibers. Interestingly, Wnt signaling is highly activated proximal to motor endplates of slow myofibers. Wnt activation induced by stabilized β-catenin inhibits muscle differentiation and promotes slow muscle determination. At the molecular level, canonical Wnt signaling induces BMP-4, which promotes expression of slow MyHC. These novel results demonstrate that interplay between Wnt and BMP signaling regulates skeletal myogenesis and muscle fiber type.

## Methods

### Plasmids

Wnts cDNA subcloned from pLNCX-HA-Wnt constructs (kindly gifted from Dr. Jan Kitajewski) to pHAN-puro retrovirus vector by PCR [27,28]. Wnt-10b and TCF-4B cDNA were obtained from Thermo Fisher Scientific Open Biosystems (Waltham, MA, USA). Super TOPFlash was kindly gifted from Dr. Moon [29]. The mouse BMP-4 shRNA pLKO.1-puro (RMM3981-9595140) was obtained from Openbiosystems. Scramble shRNA pLKO.1-puro (Addgene Plasmid #1864), MD2.G (Addgene Plasmid #12259) and psPAX2 (Addgene Plasmid #12260) were obtained from Addgene (Cambridge, MA, USA).

### C2C12 myoblasts, single myofibers and isolation of primary myoblasts

C2C12 cells are purchased from American Type Culture Collection (ATCC) (Manassas, VA, USA) and cultured in DMEM with 10% FBS and antibiotics. Cells are induced to differentiation upon 80% confluence by serum withdrawal (DMEM with 2% horse serum). Primary myoblasts were isolated from the hind limb of two- to three–month-old wild type mice as previously described [27]. Single myofibers were isolated from flexor digitorum longus (EDL) muscle by collagenase digestion. The single fibers were immediately fixed in 4% paraformaldehyde (PFA), permeabilized by Triton-X100, stained with primary antibodies for β-gal (Life Technologies, Carlsbad, CA, USA), MyHC (MF-20, Developmental Studies Hybridoma Bank [DSHB], Iowa, Iowa, USA), slow MyHC (DSHB) and FITC-conjugated α-bungarotoxin (BTX) (Sigma-Aldrich, St. Louis, MO, USA). Nuclei were counter stained with 4',6-diamidino-2-phenylindole (DAPI).

Fetal myoblasts were prepared from the legs of Myf5-Cre/ROSA26-YFP embryos at E14.5. The legs are minced and digested by collagenase, dispase and DNaseI, as previously described for satellite cells isolation [30]. After collagenase procedure, collected cells were stained by alpha7-integrin antibody and anti-mouse IgG1-Alexa648. Fetal myoblast were isolated by MoFlo (Dako, Glostrup, Denmark). Fetal myoblast were isolated by magnetic-antibody cell sorting (MACS) (Miltenyi Biotec, Bergish Gladbach, Germany). The isolated cells were incubated in culture dishes at 37°C for one hour to remove adherent cells, and the nonadherent cells were collected. The isolated fetal myoblasts cultured in DMEM/F10 medium with 20% FBS and bFGF.

### Retrovirus and lentivirus infection

To prepare ecotropic retrovirus, Phoenix-eco packaging (kindly gifted from Dr. Gally Nolan) cells were transfected with retrovirus vectors using GeneJuice (Novagen EMD Chemicals, Madison, WI, USA). Viral supernatants were harvested 30 hours post transfection and used to infect C2C12 cells in the presence of polybrene (Sigma, 8 mg/ml) for 12 hours. Infected C2C12 cells were then washed twice with phosphate-buffered saline (PBS), maintained in growth media and were selected 24 hours post-infection with puromycin (1.5 micro g/ml, Sigma). Lentivirus was packaged in 293T cells (ATCC CRL-11268) transfected with the mouse BMP-4 or scramble shRNA pLKO.1-puro plasmids in addition to the pMD2.G and psPAX2 plasmids. Lentivirus was concentrated by ultracentrifuge and resolved in PBS(−) for virus infection. Infected C2C12 and primary myoblast cells were then washed twice with PBS, and maintained in growth media.

### Gene expression analysis

Total RNAs were prepared from C2C12 and myoblast cells by TRIzol (Life Technologies). RNA samples were reverse transcribed using random hexamer and oligo dT mixed primers with SuperScriptII enzyme (Life Techonologies) according to the manufacturer’s instructions. Reverse transcription reactions were diluted (1:10) with 10 mM Tris, pH 8.0, yielding master samples of reverse-transcribed products. Real-time PCR reactions are previously described [27]. Real-time data were gathered using a system (MX4000; Agilent Technologies, Santa Clara, CA, USA) over 40 cycles (30 s at 90°C, 60 s at 58°C and 30 s at 72°C) followed by a denaturation curve from 54°C to 94°C in 30-s increments of 0.5°C to ensure amplification specificity. Threshold cycle (Ct) values were calculated with the MX4000 software (Agilent Technologies, Santa Clara, CA, USA) by using moving window aver-aging and an adaptive baseline. Fold changes, other calculations and chart plotting were performed in Microsoft Excel (Redmond, WA, USA). The sequence of PCR primers is listed in Additional file 1: Table S1.

### Animal care

Myf5-Cre [31] heterozygous mice were bred with ROSA26-YFP [32]. TCF-lacZ [33] and Ctnn1 exon3 floxed [34] mice were previously described. All mice are maintained inside a barrier facility, and experiments were performed in accordance with the University of Ottawa regulations for animal care and handling.

### Immunofluorescence staining

C2C12 cells were incubated at 37°C for two hours with 10 μM BrdU, then washed with PBS(−) and fixed with 2% PFA/PBS. The fixed cells were stained with DAPI for 15 minutes at room temperature, washed with PBS(−), and refixed with 2% PFA for 5 minutes at room temperature. The refixed cells were treated with 2N HCl for 20 minutes at room temperature (RT), neutralized by washing with 0.1 M borate buffer pH 8.5. The cells were permeablized with 0.2% Triton X-100 PBS(−), blocked with broking buffer, incubated with anti-BrdU antibody for 2 to 12 hours. After staining with the primary antibody, cells were washed with PBS(−), stained with anti-mouse IgG1-Alexa488, washed with PBS(−) and mounted on slide glass with Dako mounting buffer. Myoblasts were fixed in 4% PFA/PBS (−) for 5 minutes, blocked with 10% goat serum/PBS (−) for 10 minutes, and stained with MyHC slow, MyHC fast, MyHC pan, anti-mouse IgG1-Alexa568, anti-mouseIgG2b-Alexa648 (Life Technologies) and DAPI (Sigma). Slides were mounted in SlowFade Light antifade Kit Component A (Molecular Probes) and analyzed with a Bio-Rad confocal laser scanning microscope (model MRC-1024) (Bio-Rad Laboratories, Hercules, CA, USA).

### ALP, X-gal and NADH-TR staining

Cells were fixed 2% PFA and washed with PBS(−), then stained by alkaline phosphatase (ALP) buffer (100 mM Tris–HCl pH 9.5, 100 mM NaCl, 50 mM MgCl_2_) containing 4.5 μl nitro-blue tetrazolium chloride (NBT) and 3.5 μl 5-bromo-4-chloro-3-indolyl phosphate (BCIP) per 1 ml of ALP buffer at 37°C. Isolated whole embryos and tissue were fixed in 2% PFA for 3 hours at 4°C and permeablized with X-gal staining buffer (0.1 M phoshate buffer (pH 7.3), 2 mM MgCl_2_, 0.01% sodium deoxycholate, 0.02% Nonidet P-40) for 2 hours at 37°C and stained by X-gal staining buffer with 0.1% X-gal, 5 mM potassium ferricyanide and 5 mM potassium ferrocyanide at 37°C for 2 to 24 hours.

The isolated muscle tissues were placed directly into optimal cutting temperature (OCT) compound and frozen in deep cold isopentane with ethanol and dry ice. The muscle tissues were cut 16 μm by cryostat and dried in room temperature. The muscle sections were incubated with NADH-TR staining solution (0.8 mg/ml NADH and 1 mg/ml NBT in 50 mM Tris–HCl (pH 7.6) at 37°C. The stained muscle sections were washed with deionized water, unbound NBT was removed by acetone solution, then the sections were re-washed with deionized water and mounted with a coverslip.

### Luciferase assays

Myoblasts in 24-well plates were transfected with the plasmids indicated and 50 ng pRL-PGK using Lipofectamine (Invitrogen). Transfected cells were harvested around 24 hours after transfection, and luciferase activities in the cell extracts were measured according to the manufacturer's instructions (Promega, Fitcburg, WI, USA) in a luminometer (Microplate luminometer LB96V (EG&G Berthold Technologies, Bad Wildbad, Germany). Luciferase activities as indicated by arbitrary unit were normalized by sea urchin luciferase activities in each sample. All experiments were repeated at least three times, and the averages of more than three independent experiments with standard deviations are shown as bars [35].

### Western blots

Infected C2C12 cells were grown in 60-mm dishes, washed twice with PBS and lysed in 100 mL radioimmunoprecipitation assay (RIPA) buffer (50 mM Tris HCl, pH 7.5; 150 mM NaCl; 0.5% Nonidet P-40; 0.1% deoxycholate) containing protease inhibitor cocktail (Roche Applied Science, Penzberg, Germany) [35]. Cell extracts were collected and spun in a microcentrifuge at 13,000 rpm for 5 minutes. Total proteins (5 mg) were separated on 10% SDS-PAGE and transferred to Immobilon-P (EMD Millipore Corporation, Billerica, MA, USA). The membranes were probed with primary antibodies, followed by horseradish peroxidase (HRP)-conjugated secondary antibodies at 1:5,000 (Bio-Rad Laboratories), and developed using ECL™ Plus (GE Healthcare, Chalfont St. Giles, United Kingdam). Membranes were exposed to BIOMAX film (Eastman Kodak, Rochester, NY, USA). Primary antibodies used in this work: anti-MyoD (5.8A, BD Bioscience [San Jose, CA, USA]), anti-myogenin (F5D, DSHB), anti-MHC (MF-20, DSHB), anti-GAPDH (6C5, Life Technologies) and anti-α-tublin (Sigma-Aldrich).

## Results

### Canonical Wnt signaling is activated during fetal myogenesis and reduced in adult muscle

**Figure 1:**
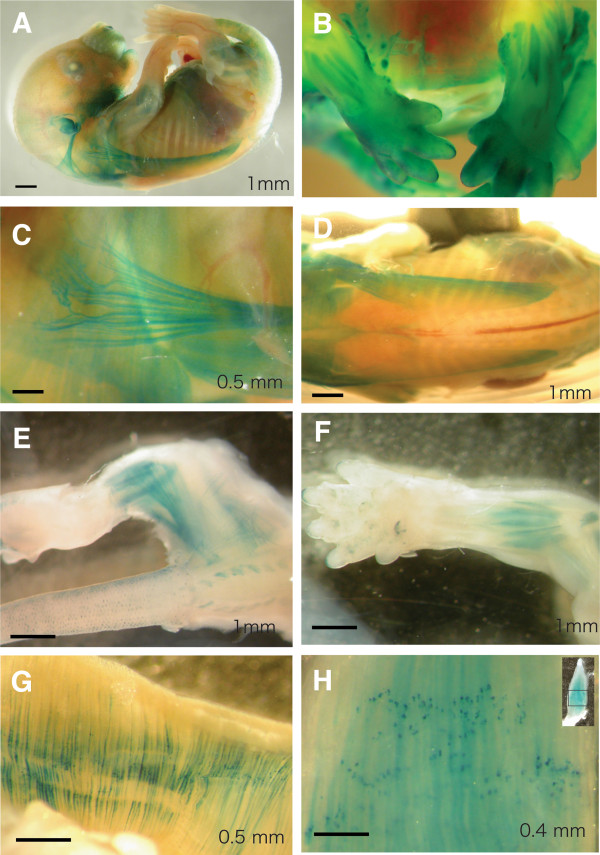
**Activation of canonical Wnt signaling in embryonic, neonatal and adult skeletal muscles.** Tcf-lacZ reporter mice were used to report activation of TCF promoter, the nuclear target of canonical Wnt signaling. X-gal staining (in blue) was used to reveal LacZ (b-gal) activity. (**A**) Whole mount staining of an E14.5 embryo. (**B**) Forelimbs and hind limbs at E14.5. (**C**) Ventral and (**D**) dorsal view of an E14.5 embryo revealing intensive staining in some muscles. (**E**) Hind limb and (**F**) forelimb at P0 (postnatal Day 0). (**G**) Diaphragm and (**H**) soleus muscles of adult mouse showing staining in a subset of myofibers with intensive signals at the neuromuscular junction area.

As the first step to investigate the function of Wnt signaling in myogenesis, we used the TCF-lacZ transgenic reporter mouse to examine the activity of the canonical Wnt signaling pathway in embryonic and adult muscles. The promoter of the LacZ transgene (encoding β-galactosidase, β-gal) contains multimerized TCF binding sites [33], a key downstream effector of canonical Wnt signaling. We analyzed β-gal activity, in embryonic and adult muscles by X-GAL staining (Figure 1). β-gal activity was detected in many muscles in E14.5 embryos (Figure 1A). Among those labeled, β-gal activity was particularly intense in both forelimb and hind limb muscles (Figure 1B), ventral body wall muscles (Figure 1C), dorsal spinotrapezius (Figure 1D) and intercostal muscles. The number of muscles labeled with X-GAL was reduced in the forelimb and hand limb in P0 fetus (Figure 1E, F). In the adult, β-gal activity was undetectable in EDL and TA, muscles that are predominantly enriched with fast type myofibers, but was readily detected in part of the diaphragm (Figure 1G) and soleus (Figure 1H), muscles that are known to be enriched with slow myofibers. These data indicate that canonical Wnt signaling is strongly activated during fetal myogenesis and declined in postnatal muscles with some residual activity in slow myofibers.

**Figure 2:**
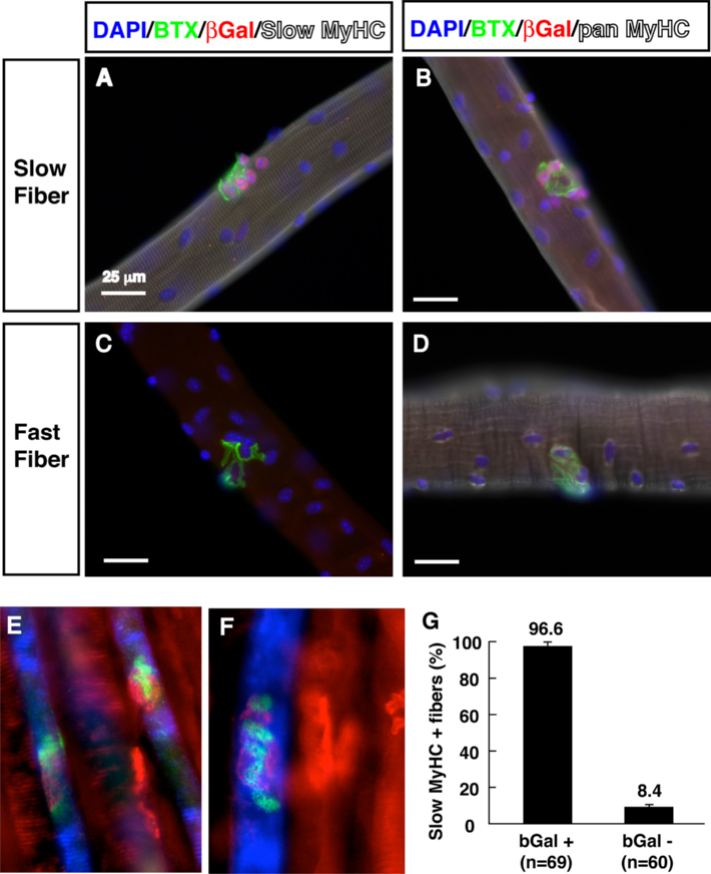
**Activation of canonical Wnt signaling in adult slow myofibers.** (**A-D**) Soleus myofibers from adult Tcf-lacZ mice were stained with antibodies for pan-myosin heavy chain (White) or slow myosin heavy chain (White), and β-gal (Red) and FITC conjugated BTX (Green). (**E-F**) Whole mount muscles co-stained with antibodies for slow (Blue) and fast (Red) myosin heavy together with beta-gal (Green) and BTX (Red). (**G**) Percentage of β-gal positive (n = 69) and negative (n = 60) myofibers that co-express slow myosin heavy chain.

Interestingly, the β-gal activity in postnatal slow muscle was especially strong at neuromuscular junctions (dark dotted staining patterns in the mid-belly of the muscles; Figure 1G-H). To further examine this phenomenon, we isolated single myofiber from the soleus muscles (containing about 50% slow and 50% fast myofibers) of Tcf-lacZ mice. The isolated single myofibers were fixed and stained with α-bungarotoxin (BTX) and β-gal antibodies, together with slow-MyHC or pan-MyHC antibodies (Figure 2A-D). Consistently, strong β-gal immunoreactivity was detected proximal to motor endplates located within BTX stained neuromuscular junctions of type I fibers (Figure 2A, B), but not in type II myofibers (Figure 2C-D). Co-labeling whole mount muscle with slow and fast myosin heavy antibody confirmed the specific activation of β-gal in the in slow myofibers (Figure 2E, F). Overall, 97% of the β-gal^+^ myofibers co-expressed the slow MyHC, where only 8% of the β-gal^-^ myofibers co-expressed slow MyHC (Figure 2G). These data indicate that canonical Wnt signaling is highly activated at the neuromuscular junction area specifically in slow myofibers of adult skeletal muscles.

### Canonical Wnt signaling promotes formation of slow myofibers *in vivo*

To confirm the role of canonical Wnt signaling in muscle fiber type specification *in vivo*, we took advantage of the Ctnnb^lox(ex3)^ transgenic mice in which the exon 3 of *β-catenin* (*Ctnnb*) gene flanked by LoxP sites [34]. The exon 3 encodes serine and threonine residues that are normally phosphorylated GSK3β, leading to the proteasomal degradation of β-catenin. Upon Cre-mediated excision of *Ctnnb* exon 3, β-catenin^ΔEx3^ is prevented from degradation (stabilized) and, therefore, constitutively active.

**Figure 3:**
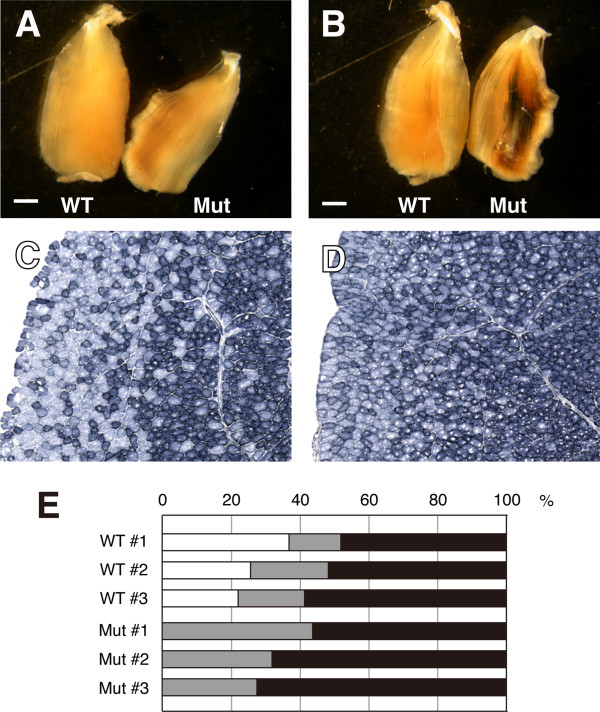
**Muscle-specific constitutive activation of canonical Wnt signaling promotes oxidative myofiber phenotype.** The MCK-Cre/Ctnnb^lox(ex3)^ mice were used to express constitutively active β-catenin^Δex3^, which mimics canonical Wnt signaling. The MCK promoter-drive Cre expression limits Wnt activation only in mature skeletal muscles. (**A-B**) X-Gal staining (blue signal) of whole mount TA muscles of Ctnnb^lox(ex3)^/Tcf-LacZ (WT) and MCK-Cre/Ctnnb^lox(ex3)^/Tcf-LacZ (Mut) mice. Blue signal, indicative of canonical Wnt signaling, is only detectable in the Mut TA muscles (**A** and **B** represent the dorsal and ventral view of the same muscles). (**C-D**) NADH-TR staining of TA muscle sections from the WT (**C**) and Mut (**D**) mice. (**E**) Percentage of high oxidative (black), middle oxidative (gray) and low oxidative (white) myofibers of WT and Mut mice (n = 3, each). Scale bar: 1 mm.

We first used Myf5-Cre to induce β-catenin^ΔEx3^ expression in myogenic progenitor cells. Myf5 is an early myogenic commitment marker during embryonic myogenesis [36]. Myf5-Cre/Ctnnb^lox(ex3)^ mice die at E15.5 with extremely reduced muscle mass (data not shown), thus precluding further analysis of myofiber types. We next used MCK-Cre to drive β-catenin^ΔEx3^ expression only in differentiated muscle cells. As expected, we detected increased activation of canonical Wnt signaling *in vivo* in transgenic mice carrying MCK-Cre, Ctnnb^lox(ex3)^ and TCF-LacZ alleles. β-gal activity was detectable in TA muscles of the MCK-Cre/Ctnnb^lox(ex3)^/TCF-LacZ mice, but not the Ctnnb^lox(ex3)^ /Tcf-lacZ littermate controls (Figure 3A, B).

In addition, we analyzed the oxidative activity of skeletal muscle myofibers using NADH-tetrazolium (NADH-TR) staining. Strikingly, the high and middle oxidative myofibers were increased in TA muscles of MCK-Cre/ Ctnnb^lox(ex3)^ mice (n = 3, 65.4 ± 8.4% and 34.6 ± 8.4%, respectively) compared to the same muscle of wild type littermates (n = 3, 52.7 ± 5.3% and 19.0 ± 3.8%, respectively) (Figure 3C-E). By contrast, the number of the low oxidative myofibers was increased in wild type TA muscles (n = 3, 28.3 ± 7.7%) compared relative to the mutant littermate (Figure 3C-E). As slow muscles are known to contain mainly oxidative myofibers and fast muscles are mainly low oxidative and glycolytic [19], these data provide *in vivo* evidence that canonical Wnt signaling promotes slow myofiber phenotype in the postnatal skeletal muscle.

### Canonical Wnt signaling inhibits proliferation of C2C12 and primary myoblasts

To identify the Wnt molecules that activate the canonical Wnt signaling pathway in muscle, we co-transfected candidate Wnt plasmids with the TCF/LEF reporter Super TopFlash. We found that Wnt-1 and Wnt-3a strongly activated (>400 times increase in luciferase activity), Wnt-2 and Wnt-10b moderately activated (>20 times increase in luciferase activity), and Wnt-2b and Wnt-4 weakly activated (>2 times increase in luciferase activity) the canonical Wnt reporter (Additional file 2: Figure S1A). Other Wnts (Wnt-5a, Wnt-5b, Wnt-6, Wnt-7a, Wnt-7b, Wnt-10a, Wnt-11) had no effect on the activation of the Super TopFlash reporter (Additional file 2, Figure S1A). We, therefore, used Wnt-1 and Wnt-3a to activate the canonical Wnt signaling in the following studies.

We next examined the function of both canonical and non-canonical Wnt signaling in cultured C2C12 myoblasts using retrovirus expressing various Wnts. Compared to mock controls, C2C12 cells overexpressing canonical Wnts (Wnt-1, -2, -2b, -3a, -10b) had a significantly decreased cell number after 96 h in culture (Additional file 2: Figure S1B). By contrast, C2C12 cells overexpressing non-canonical Wnts (Wnt-5a, -5b, -6, -7b, -11) had a moderately increased cell number compared to the control treated cells (Additional file 2: Figure S1B).

A reduced cell number in C2C12 cells overexpressing Wnt-1, -2, -2b, -3a and -10b indicates that canonical Wnt signaling inhibits cell proliferation. To confirm this, we analyzed the expression of Ki-67 (Additional file 2: Figure S1 C-E), a nuclear antigen specifically expressed in S, G2 and M phase cells. The Wnt-1 and -3a expressing C2C12 cells had decreased Ki-67 expression (Additional file 2: Figures S1 D-E, and 4I). In addition, we examined incorporation of BrdU, a thymidine analog that is incorporated into proliferating cells during the S phase. Proliferating C2C12 cells were incubated with BrdU for one hour and then fixed for BrdU staining (Additional file 2: Figure S1F-H). Consistently, BrdU incorporation was reduced in the Wnt-1 and -3a expressing C2C12 cells (Additional file 2: Figure S1G-H, J). Furthermore, we analyzed proliferation of skeletal muscle-derived primary myoblasts cultured with recombinant Wnt-3a protein (50 ng/ml) for 24 hours. Both Ki-67 expression (Additional file 3: Figure S2A-C) and BrdU uptake (Additional file 3: Figure S2D-F) were decreased in the presence of Wnt-3a protein. These results indicate that canonical Wnt signaling suppressed the proliferation of C2C12 cells and adult primary myoblasts.

**Figure 4:**
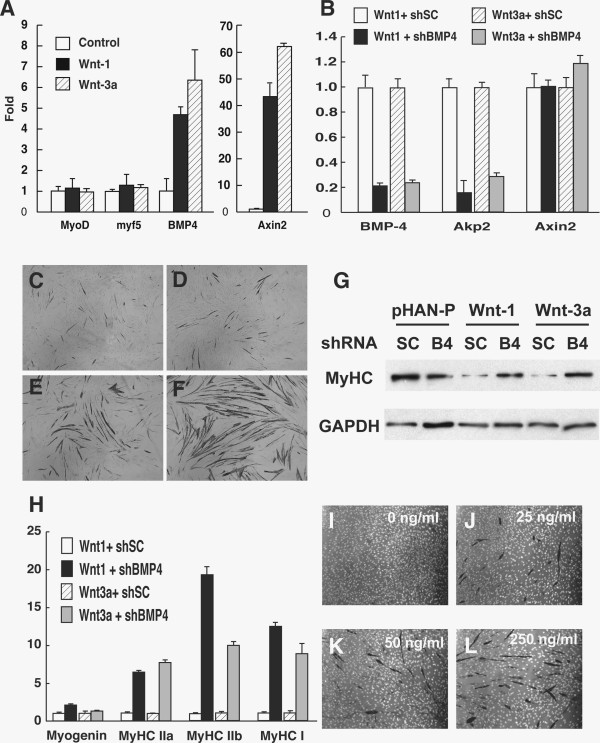
**Canonical Wnt signaling induces BMP-4 in C2C12 myoblasts.** (**A**) qPCR analysis of the relative expression of various genes in C2C12 myoblasts overexpressing Wnt-1 and Wnt-3a under growth condition. (**B**) *BMP-4* knockdown reduced the expression of *alkaline phosphatase* (*Akp2*) but not the *Axin2* gene. (**C-F**) *BMP-4* shRNA rescued the myogenic differentiation of Wnt-1 and Wnt-3a infected C2C12 cells. (C-D) Wnt-1 and Wnt-3a overexpressing myoblasts treated with Scramble shRNA; (E-F) Wnt-1 and Wnt-3a overexpressing myoblasts treated with *BMP-4* shRNA. Black signaling is myosin heavy chain antibody staining reacted with 3, 3'-diaminobenzidine (DAB) substrate. (**G**) Western blotting showing myosin heavy chain protein expression after *BMP-4* shRNA treatment. (**H**) qPCR analysis of myosin heavy chain gene expression. (**I-L**) BMP-4 antagonist Noggin-Fc dose-dependently rescued the myogenic differentiation of Wnt-3a infected C2C12 cells. The myosin heavy chain was labeled in black and nuclei were labeled in white.

To test if Wnt signaling has any effect on myogenic differentiation, we induced C2C12 myoblasts to differentiate. Upon serum withdrawal, control myoblasts exited the cell cycle and fused to form myotubes with uniform morphology (Additional file 4: Figure S3A). By contrast, Wnt-1 and Wnt-3a expressing C2C12 cells formed very few myosin heavy chain (MyHC) positive myotubes (Additional file 4: Figure S3B-C). In addition, these myotubes were morphologically abnormal: they were short and chubby (Additional file 4: Figure S3B-C). As previously reported, non-canonical Wnt7a overexpression led to the formation of large myotubes resembling the muscle hypertrophy phenotype (Additional file 4: Figure S3D). We further examined by Western blotting the expression of two myogenic differentiation markers, myogenin and MyHC. Compared to the control, Wnt-1 and -3a robustly inhibited the expression of Myogenin and MyHC at 48 hours, 72 hours and 96 hours post-induction of differentiation (Additional file 4: Figure S3E). Intriguingly, the non-canonical Wnt-7a not only increased Myogenin and MyHC protein levels, but also induced their earlier expression at 24 hours (Additional file 4: Figure S3E). However, recombinant Wnt-3a protein at 50 ng/ml had no effect on the differentiation of neither C2C12 myoblasts (not shown) nor fetal primary myoblasts (see Figure 4H). Thus, these data indicate that prolonged high-level constitutive activation of Wnt-1 and Wnt-3a in C2C12 cells suppresses myogenic differentiation.

### Canonical Wnt signaling activates BMP signaling in C2C12 myoblasts

The reduced myogenic differentiation of Wnt-1 and Wnt-3a overexpressing C2C12 myoblasts prompted us to analyze the alternative differentiation fate of these cells. Previous studies show that C2C12 cells can also differentiate into osteogenic lineage [37], which express ALP. Surprisingly, Wnt-1, Wnt-3a and Wnt-10b overexpression strongly induced ALP immunochemical signals (Additional file 5: Figure S4A-F). By contrast, expression of non-canonical Wnt-5a and Wnt-7a did not affect ALP expression (Additional file 5: Figure S4D-E). Quantitative analysis confirms that Wnt-1 and Wnt-3a increased ALP enzyme activity by 15- and 30-fold, respectively (Additional file 5: Figure S4G).

To examine if Wnt-induced ALP activity was dependent on β-catenin, which mediates canonical Wnt signaling, we transduced C2C12 cells with a dominant negative TCF-4b (DN-TCF-4b) that lacks the β-catenin binding site. As expected, DN-TCF-4b suppressed both Wnt-1 and Wnt-3a induced ALP activity in C2C12 cells (Additional file 5: Figure S4H). These data indicate that canonical Wnt signaling induces ALP expression via a β-catenin/TCF-dependent pathway in C2C12 cells.

That canonical Wnt signaling induces osteogenic ALP expression suggests a potential interaction between Wnt and BMP signaling pathways. We first examined BMP-4 gene expression using quantitative RT-PCR (qPCR) given its role in osteogenesis. Indeed, *BMP-4* mRNA expression was increased by more than five-fold in Wnt-1 and Wnt-3a overexpressing C2C12 cells (Figure 4A). In comparison, *Myf5* and *MyoD* expression was not affected by Wnt-1 and Wnt-3a (Figure 4A). To ensure that canonical Wnt signaling is involved in the induction of BMP-4, we examined Axin2, a transcriptional target of β-catenin and canonical Wnt signaling [38]. Wnt-1 and Wnt-3a overexpression led to over 40X increase in the expression *Axin2* (Figure 4A).

**Figure 5:**
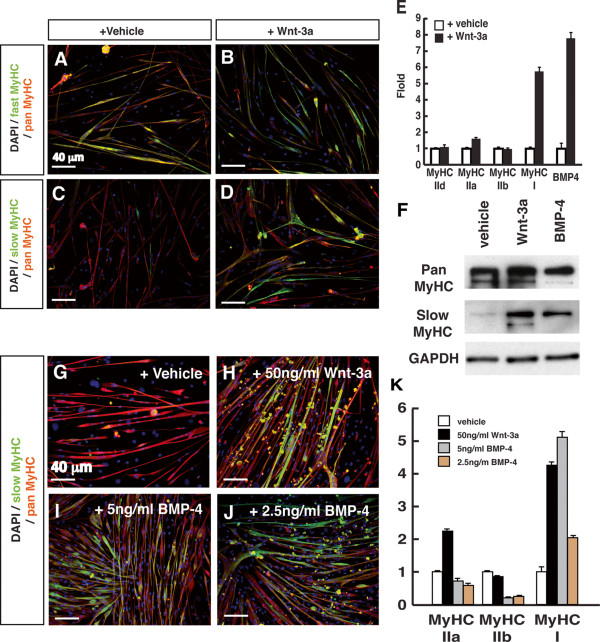
**Wnt-3a induces slow myosin heavy chain expression in fetal myoblasts via BMP-4.** Fetal myoblasts were isolated by fluorescence-activated cell sorting (FACS) from E14.5-15.5 embryos of Myf5-Cre/Rosa26-YFP mice. FACS isolated fetal myoblasts were cultured and induced to differentiate for three days. (**A-D**) Immunofluorescence showing slow myosin heavy chain (green) expression in myotubes treated with vehicle control medium or Wnt-3a recombinant protein (50 ng/ml). (**E**) Relative expression of myosin heavy chain and BMP-4 genes based on qPCR analysis. (**F**) Western blotting showing slow myosin heavy chain protein expression. (**G-J**) Immunofluorescence showing slow- (green) and pan- (red) myosin heavy chain expression in myotubes treated with vehicle control medium, Wnt-3a, and BMP-4 recombinant protein at concentration shown. (**K**) qPCR analysis showing relative expression of myosin heavy chain isoform genes. Scale bars: 40 μm.

We next asked if BMP-4 is necessary for pro-osteogenic effect of canonical Wnt signaling. We used lentiviral shRNA mediated knockdown of *BMP-4* in C2C12 cells. This approach resulted in nearly 80% reduction of *BMP-4* transcripts (Figure 4B). Importantly, BMP-4 knockdown reduced Wnt-1 and Wnt-3a induced *Akp4* (ALP gene) expression by more than 70% (Figure 4B). By contrast, *Axin2* mRNA levels were not decreased following knockdown of *BMP-4* (Figure 4B), suggesting that *BMP-4* signaling does not affect canonical Wnt signaling.

We further investigated if the anti-myogenic effect of canonical Wnt signaling is mediated by BMP-4 using the same shRNA knockdown approach. In the control groups (scrambled shRNA), there were only a few MyHC positive myotubes in the C2C12 cells overexpressing Wnt-1 (Figure 4C) and Wnt-3a (Figure 4C). Knockdown of *BMP-4* remarkably increased the numbers of MyHC positive myotubes in Wnt-1 (Figure 4E) and Wnt-3a (Figure 4F) expressing C2C12 cells. Consistent with this observation, *BMP-4* shRNA rescued MyHC protein expression in Wnt-1 and Wnt-3a overexpressing C2C12 cells (Figure 4G). Moreover, *BMP-4* shRNA increased the mRNA levels of *Myogenin*, *MyHC-IIa*, *MyHC*-*IIb* and *MyHC*-*I* (Figure 4H). To confirm the above observations, we used recombinant Noggin protein, an antagonist of BMP, to block BMP activity. Noggin dose-dependently increased the number of MyHC positive myotubes in Wnt-3a expressing C2C12 cells (Figure 4I-L). These data provide compelling evidence that canonical Wnt signaling inhibits myogenic differentiation through inducing BMP-4 signaling.

### Canonical Wnt signaling induces slow MyHC expression through BMP-4

To directly examine the function of canonical Wnt signaling in muscle fiber type specification, we isolated embryonic myoblasts from E14.5 embryos by FACS. We employed positive selection for Myf5 and α7-integrin expression of myogenic cells from *Myf5-Cre/ROSA-YFP* embryos (Additional file 6: Figure S5). The purity of isolated fetal myoblasts was confirmed by immunostaining for Pax7 and desmin (Additional file 6: Figure S5). Embryonic myoblasts were cultured for one day before being induced to differentiate with or without the addition of Wnt-3a protein (50 ng/ml). In the absence of Wnt-3a, embryonic myoblasts differentiated into myotubes that expressed fast MyHC (Figure 5A), but not slow MyHC (Figure 5C) in agreement with previous studies [39]. By contrast, Wnt-3a treated fetal myoblasts differentiated into myotubes that expressed both fast (Figure 5B) and slow MyHC (Figure 5D).

To confirm this result, we analyzed *MyHC-I* mRNA expression by qPCR. The *MyHC-I* mRNA level was up-regulated 5.5-fold by Wnt-3a compared to control vehicle treatment (Figure 5E). Wnt3a also robustly induced the expression of slow MyHC-I at the protein level (Figure 5F). These results indicate that canonical Wnt signaling induces slow MyHC expression in fetal myoblasts.

As canonical Wnt signaling induced BMP-4, we further examined the role of BMP-4 in muscle fiber type specification. Consistent with our previous results in C2C12 cells (Figure 4A), Wnt-3a treatment of embryonic myoblasts induced a seven-fold increase in *BMP-4* mRNA expression (Figure 5E). Next, we added recombinant BMP-4 to fetal myoblast cultures during differentiation. In the control treated with vehicle medium, newly formed MyHC^+^ myotubes seldom expressed slow MyHC after three days of differentiation (Figure 5G). In the presence of 2.5 to 5 ng/ml BMP-4, slow-MyHC^+^ myotubes were abundantly visible (Figure 5I-J). The level of slow MyHC immunofluorescence induced by 5 ng/ml BMP-4 is similar to that induced by 50 ng/ml Wnt-3a (Figure 5H), suggesting BMP-4 more potently induces slow MyHC expression. Western blotting showed that the slow MyHC protein expression level was indeed increased in the presence of BMP-4 (Figure 5F). In addition, qPCR analysis indicated that BMP-4 not only induced the slow *MyHC-I* gene expression, but also robustly suppressed the fast *MyHC-IIb* gene expression (Figure 5K). Collectively, these results indicate that canonical Wnt signaling acts through BMP-4 to induce slow MyHC expression during embryonic myoblast differentiation.

## Discussion

In this study, we use genetic, cell culture and molecular biology approaches to dissect the function of canonical Wnt signaling in myogenic differentiation and skeletal myofiber types. We show that the canonical Wnt signaling is most active during perinatal myogenesis and only activated in slow myofibers with high activity at the neuromuscular junction area in mature muscles. Constitutive activation of β-catenin, the canonical Wnt signaling effector, leads to impaired myogenesis and an increased proportion of oxidative myofibers in the postnatal muscles. Importantly, Wnt-1 and Wnt-3a mediated downstream signaling activates BMP-4, which inhibits the overall proliferation of myoblasts and promotes myogenic differentiation towards slow muscle phenotype. These results establish a novel interaction between Wnt and BMP signaling that regulates muscle fiber type specification and maintenance.

The TCF-LacZ reporter mouse has been widely used in reporting activation of canonical Wnt signaling in various tissues/cells [11,40]. Strong LacZ expression in specific muscles during embryonic and fetal myogenesis indicates activation of canonical Wnt signaling. Interesting, several muscles (spinotrapezius, body wall muscle and diaphragm) with high β-gal activity are known to be enriched with slow myofibers [19]. These results suggest a role of Wnt signaling in slow muscle generation and maintenance. Our *in vivo* results are consistent with previous studies in chick and fish embryos, in which canonical Wnt signaling was shown to promote slow muscle fate [20-22].

Our analysis of canonical Wnt signaling in adult mature muscles reveal several interesting points. First, β-gal activity is only detectable in muscles known to contain slow myofibers. This result confirms our observation in the developing embryonic muscles. Second, in contrast to embryonic muscle, where β-gal activity is evenly distributed within myofibers, the highest β-gal activity was within the slow myofibers was proximal to the motor endplate (Figure 1G-H). This observation suggests that whereas in embryonic muscle the Wnt molecules are released from surrounding tissues [5], Wnt signaling in adult slow myofibers is most likely initiated by Wnt molecules from motor neurons that innervate these myofibers. In support of the notion that the motor neuron supplies Wnt molecules, we found that β-gal immunoreactivity was no longer detectable in slow myofibers after three days of suspended culture *in vitro* in the absence of neural innervation (data not shown).

Previous studies demonstrate that Wnt molecules released by motor neurons play key roles in the development of neuromuscular junctions. Specifically, interaction of Wnt and LRP is necessary for clustering of postsynaptic acetylcholine receptors (AchR) [24]. Our new results demonstrate that Wnt signaling is further required for the maintenance of neuromuscular junction in slow myofibers. Future study is needed to examine the functional significance of Wnt signaling in slow versus fast muscle fibers and identify the Wnt molecules released by slow and fast motor neurons.

Using Cre-inducible transgenic mice that express stabilizes β-catenin, we investigated the role of canonical Wnt signaling in embryonic myogenesis and postnatal muscle maintenance. When Myf5-Cre is used as the driver mouse, which is expressed in embryonic myogenic progenitors, we detected abnormal muscle development and perinatal lethality. This observation is consistent to recent studies using Pax7-Cre or Myogenin-Cre to stabilize β-catenin, which also results in lethality at P0 [23,41]. In these studies, constitutive activation of β-catenin in the myogenic progenitors and committed myocytes resulted in a shift of fetal myofibers to slow muscle phenotype, and reduced myofiber size. However, the perinatal lethality of the Myf5-Cre, Myogenin-Cre and Pax7-Cre drive β-catenin activation precludes analysis of postnatal muscles. Using the Mck-Cre/ctnnb^Lox(ex3)^ mice, we found that adult muscles indeed have higher canonical Wnt activity based on the TCF-LacZ reporter assay. This verifies the utility of our mouse model.

Importantly, we found the adult fast (TA) muscles exhibited features of slow muscle phenotype as increased oxidative capacity. We also examined myosin heavy chain expression by immunohistochemistry but did not find any overt changes in myofiber types (data not shown). This result suggests that although Wnt signaling affects metabolic properties in the adult muscles, it is not sufficient to switch myosin heavy chain expression in the adult. This is expected since other factors, such as hormones and neural activity, can also influence myosin expression [19]. Together, our *Mck-Cre/ctnnb*^*Lox(ex3)*^ model bypasses the premature lethality and provides novel insights of canonical Wnt signaling in regulating the oxidative capacity of adult muscles.

The observation that C2C12 cells overexpressing Wnt-1 and Wnt-3a exhibited reduced proliferation and myogenic differentiation is quite intriguing. It could suggest that canonical Wnt/β-catenin inhibits the proliferation and differentiation of myogenic cell lineages. This possibility would explain the reduced muscle mass phenotypes of the Myf5-Cre, Myogenin-Cre and Pax7-Cre induced stabilized β-catenin mice [23,41]. Alternatively, the result may also suggest that expression of Wnt-1 and Wnt-3a in the cell, independent of Frizzled receptor activation, is detrimental to cell growth and differentiation.

Interestingly, embryonic and fetal myoblasts seem to have different responses to canonical Wnt signaling [23], suggesting that the role of Wnt signaling, even in the same cell lineage, is also context dependent. Consistent with this notion, we show that the growth and differentiation of fetal primary myoblasts are not inhibited by recombinant Wnt-3a protein in culture. The osteogenic fate choice of Wnt-1 and Wnt-3a overexpressing C2C12 cells is in line with a recent report demonstrating fibroblastic lineage differentiation of satellite cells in response to high level of systemic Wnt molecules [18]. Thus, the observed effect of Wnt-1 and Wnt-3 in myogenic cell proliferation and differentiation is largely consistent with information in the literature.

We discovered a novel interaction between Wnt and BMP signaling in myoblasts. Bone morphogenetic proteins (BMPs) are multi-functional proteins belonging to the transforming growth factor beta (TGFβ) superfamily. In zebrafish and frogs, BMP signaling inhibits the differentiation of muscle precursors in the dermomyotome and controls the number of myogenic cells [42,43]. During late myogenesis of mice, BMP signaling regulates the number of fetal myoblasts and satellite cells [44]. This action is through preventing the premature activation of MyoD while maintaining Pax3 expression. Therefore, BMPs may function to establish a sufficient number of myogenic progenitors before terminal differentiation.

Our cell culture results indicate that Wnt signaling induces BMP4, and BMP4 inhibition rescues the inhibitory effect of Wnt-1 and Wnt-3a on myogenesis. This result is in line with the above results *in vivo*. We further identify an unexpected role for BMP-4 in promoting slow muscle fate during fetal myogenesis. It is important to mention that low concentrations (1 to 5 ng/ml) of BMP-4 protein were used in our study. Non-physiological, high concentrations of BMP will probably generate completely different effects [45]. Future studies should illustrate how BMP signaling regulates myosin gene expression.

Interestingly, in *Drosophila* larval neuromuscular junctions, retrograde BMP signaling controls synaptic growth [46]. The muscle-derived BMP modulates cytoskeletal dynamics and structural changes at presynaptic terminals. This forms a feedback system in which canonical Wnt molecules secreted from motor neurons not only induce formation of neuromuscular junctions, but also activate BMP-4 expression in the muscle. The muscle derived BMP-4 subsequently promotes development of presynaptic motor neuron terminals. Indeed, β-catenin stabilization in skeletal muscles (not limited to the neuromuscular junction area) results in increased motor axon number and excessive intramuscular nerve defasciculation and branching [41]. Taken together, our experiments have identified a novel interaction between canonical Wnt and BMP signaling that plays a role in myofiber type specification.

## Conclusion

Our study demonstrates that canonical Wnt-signaling controls the development of skeletal muscles via BMP-4 expression. High concentrations of BMP-4 have been previously established to inhibit myogensis and induce osteogenesis. We found that isolated fetal myoblasts do not normally form slow myofibers during myogenic differentiation *in vitro*. Strikingly, canonical Wnt-signaling induced low level BMP-4 expression that act to induce slow myofibergenesis. Therefore, we conclude that canonical Wnt and BMP signaling plays a hitherto unappreciated role in myofiber type specification during fetal myogenesis.

## Abbreviations

BMP: Bone morphogenetic protein; TCF: T-cell factor; LEF: Lymphoid enhancer-binding factor; Pax: Paired box protein; FBS: Fetal bovine serum; bFGF: Basic fibroblast growth factor; PFA: Paraformaldehyde; PGK: Phosphoglycerate kinase; GSK: Glycogen synthase kinase; MCK: Muscle creatine kinase; BrdU: Bromodeoxyuridine.

## Competing interests

The authors declare no competing interests.

## Authors’ contributions

KK and MAR designed the research and wrote paper. SK performed the histology and tissue staining, and helped with paper writing. TMM provided the Ctnn1 exon3 floxed mice. All authors read and approved the final manuscript.

## Supplementary Material

Additional file 1: Table S1 The sequence of PCR primers for qPCR analysis is listed. Click here for file

Additional file 2: Figure S1 Canonical Wnt signaling inhibits the growth and proliferation of C2C12 myoblast cells. (**A**) Relative luciferase activity of C2C12 cells overexpressing various Wnt genes together with the SuperTop Flash reporter plasmid. (**B**) Graph of C2C12 cells transduced with retroviral Wnt plasmids. (**C-E**) The Ki-67 antibody staining and (**F-H**) BrdU incorporation of control, Wnt-1 and Wnt-3a transduced cells. (**I**) Percentage of Ki-67 positive cells in control, Wnt-1 and Wnt-3a transduced cells. (**J**) Percentage of BrdU incorporated cells in Control, Wnt-1 and Wnt-3a retrovirus infected C2C12 cells. Click here for file

Additional file 3: Figure S2 Wnt-3a inhibits proliferation of adult primary myoblasts. (**A-B**) Ki-67 antibody staining of primary myoblasts treated with vehicle control (**A**) and Wnt-3a recombinant protein (50 ng/ml) (**B**). (**C**) Percentage of Ki67 positive cells. (**D-E**) BrdU incorporation of primary myoblast cells treated with control (**D**) and Wnt-3a protein (E). (**F**) Percentage of cells incorporated BrdU. Click here for file

Additional file 4: Figure S3 Overexpression of Wnt-1 and Wnt-3a inhibits the myogenic differentiation of C2C12 myoblasts. (**A-D**) Immunofluorescence showing sarcomeric myosin heavy chain protein expression in C2C12 cells transduced with retrovirus overexpressing (**A**) Mock, (**B**) Wnt-1, (**C**) Wnt-3a and (**D**) Wnt-7a after four days of induced myogenic differentiation. (**E**) Western blotting analysis showing expression of myosin heavy chain (MyHC), myogenin and GAPDH in control, Wnt-1, Wnt-3a and Wnt-7a overexpression C2C12 myoblasts at different time points after induced to differentiate. GAPDH: Glyceraldehyde 3-phosphate dehydrogenase. Scale bar: 100 μm. Click here for file

Additional file 5: Figure S4 Canonical Wnts (−1, -3a and 10b) induces alkaline phosphatase (ALP) activity in C2C12 cells. (**A-F**) ALP staining (Purple signal) of C2C12 cells transduced retrovirus expressing empty vector (Mock) or Wnt vectors as indicated. (**G**) Relative ALP activity. (**H**) The dominant negative (DN) TCF-4 suppressed ALP activity in Wnt-1 and Wnt-3a expressing C2C12 cells. Click here for file

Additional file 6: Figure S5 Isolation of fetal myoblasts from E14.5 embryos of Myf5-Cre/ROSA26-YFP mice by fluorescence activated cell sorting (FACS). (A-C) YFP expression in whole embryo (**A**), forelimb (**B**) and hindlimb (**C**) of E14.5-15.5 Myf5-Cre/ROSA26-YFP embryo. (**D**) Strategy for isolating fetal myoblasts by FACS. Whole limbs of Myf5-Cre/ROSA26-YFP embryos were minced and digested by collagenase and dispase. Single cells were stained with alpha7-integrin antibody and selected by YFP and alpha7-integrin expression. The sorted YFP and alpha7-integrin double positive cells were stained with antibody for Pax7 and desmin, makers of fetal myoblasts (nuclei were counterstained by DAPI in blue). Click here for file
