# Supplementary material for: Canonical Wnt signaling induces BMP-4 to specify slow myofibrogenesis of fetal myoblasts
Source: Skelet Muscle. 2013 Mar 5;3:5. doi: 10.1186/2044-5040-3-5 (PMC3602004; doi:10.1186/2044-5040-3-5)
Supplement: Additional file 1: Table S1 — The sequence of PCR primers for qPCR analysis is listed. [file 2044-5040-3-5-S1.docx]

**Supplemental Table 1**

| Gene | Forwod primer | Reverse primer | PCR product size | NCBI Reference Number |
| --- | --- | --- | --- | --- |
| Myf-5 | acagcagctttgacagcatc | agctggacacggagctttta | 74 | NM_008656.3 |
| MyoD | cattccaacccacagaacct | caagccctgagagtcgtctt | 80 | NM_010866.1 |
| Myogenin | actcccttacgtccatcgtg | caggacagccccacttaaaa | 175 | NM_031189.2 |
| BMP-4 | ccgaatgctgatggtcgttt | cctgaatctcggcgacttttt | 104 | NM_007554.2 |
| Akp2 | aatcggaacaacctgactgacc | tccttccaccagcaagaagaa | 102 | NM_007431.2 |
| Axin2 | cgctcgggtttgtgttaagt | gtcaacgctctgccctacac | 92 | NM_015732.4 |
| MyHC I | agtcccaggtcaacaagctg | ttccacctaaagggctgttg | 145 | NM_080728.2 |
| MyHC IIa | agtcccaggtcaacaagctg | gcatgaccaaaggtttcaca | 130 | NM_001039545.2 |
| MyHC IIb | agtcccaggtcaacaagctg | tttctcctgtcacctctcaaca | 116 | NM_010855.2 |
| MyHC IId | agtcccaggtcaacaagctg | cacattttgctcatctctttgg | 113 | NM_030679.1 |
| α-actin | atgctccccgggctgtat | cataggagtccttctgacccattc | 87 | NM_007393.1 |
