# Supplementary figures and images for: Canonical Wnt signaling induces BMP-4 to specify slow myofibrogenesis of fetal myoblasts
Source: Skelet Muscle. 2013 Mar 5;3:5. doi: 10.1186/2044-5040-3-5 (PMC3602004; doi:10.1186/2044-5040-3-5)

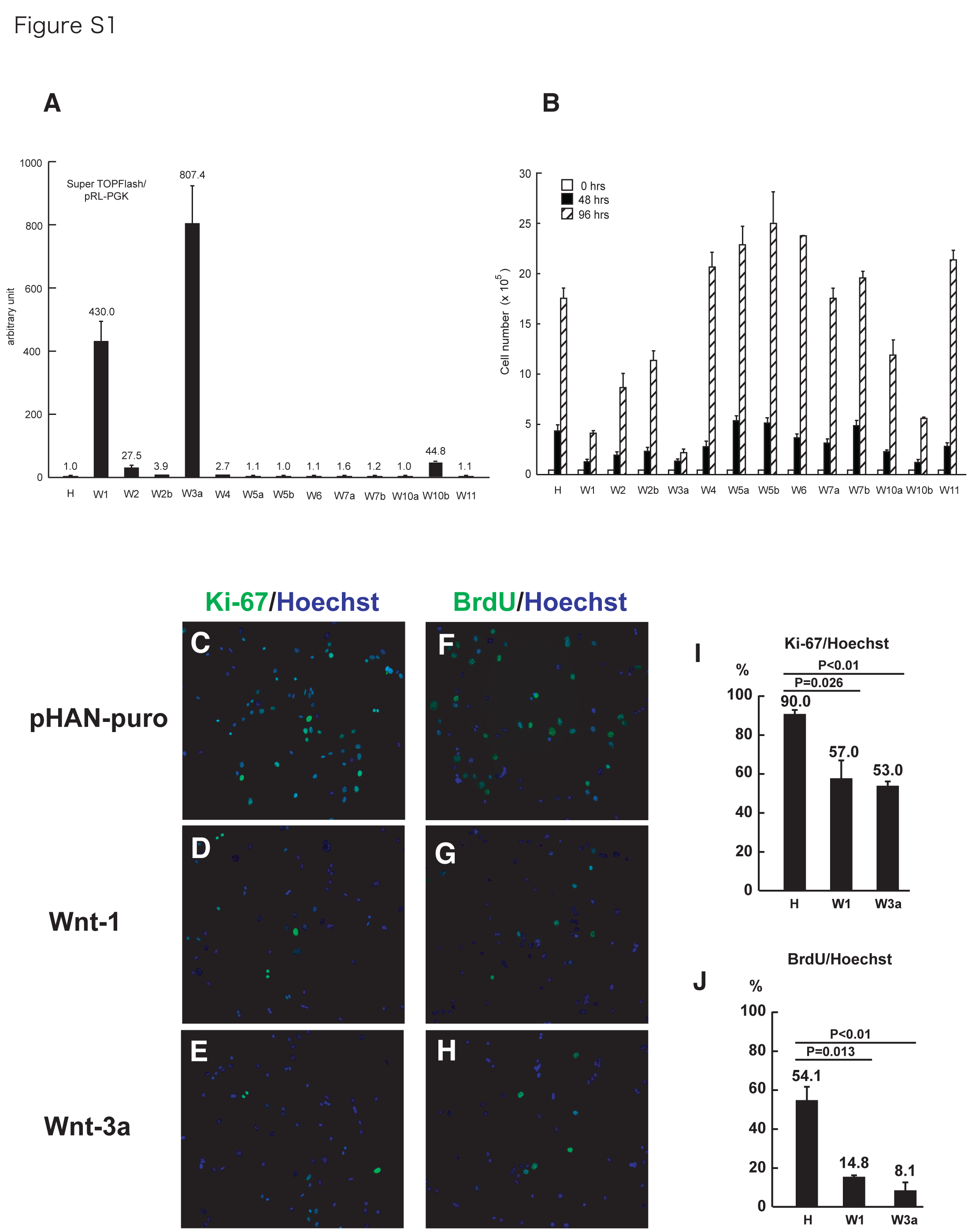

Supplement: Additional file 2: Figure S1 — Canonical Wnt signaling inhibits the growth and proliferation of C2C12 myoblast cells. (A) Relative luciferase activity of C2C12 cells overexpressing various Wnt genes together with the SuperTop Flash reporter plasmid. (B) Graph of C2C12 cells transduced with retroviral Wnt plasmids. (C-E) The Ki-67 antibody staining and (F-H) BrdU incorporation of control, Wnt-1 and Wnt-3a transduced cells. (I) Percentage of Ki-67 positive cells in control, Wnt-1 and Wnt-3a transduced cells. (J) Percentage of BrdU incorporated cells in Control, Wnt-1 and Wnt-3a retrovirus infected C2C12 cells. [file 2044-5040-3-5-S2.tiff]

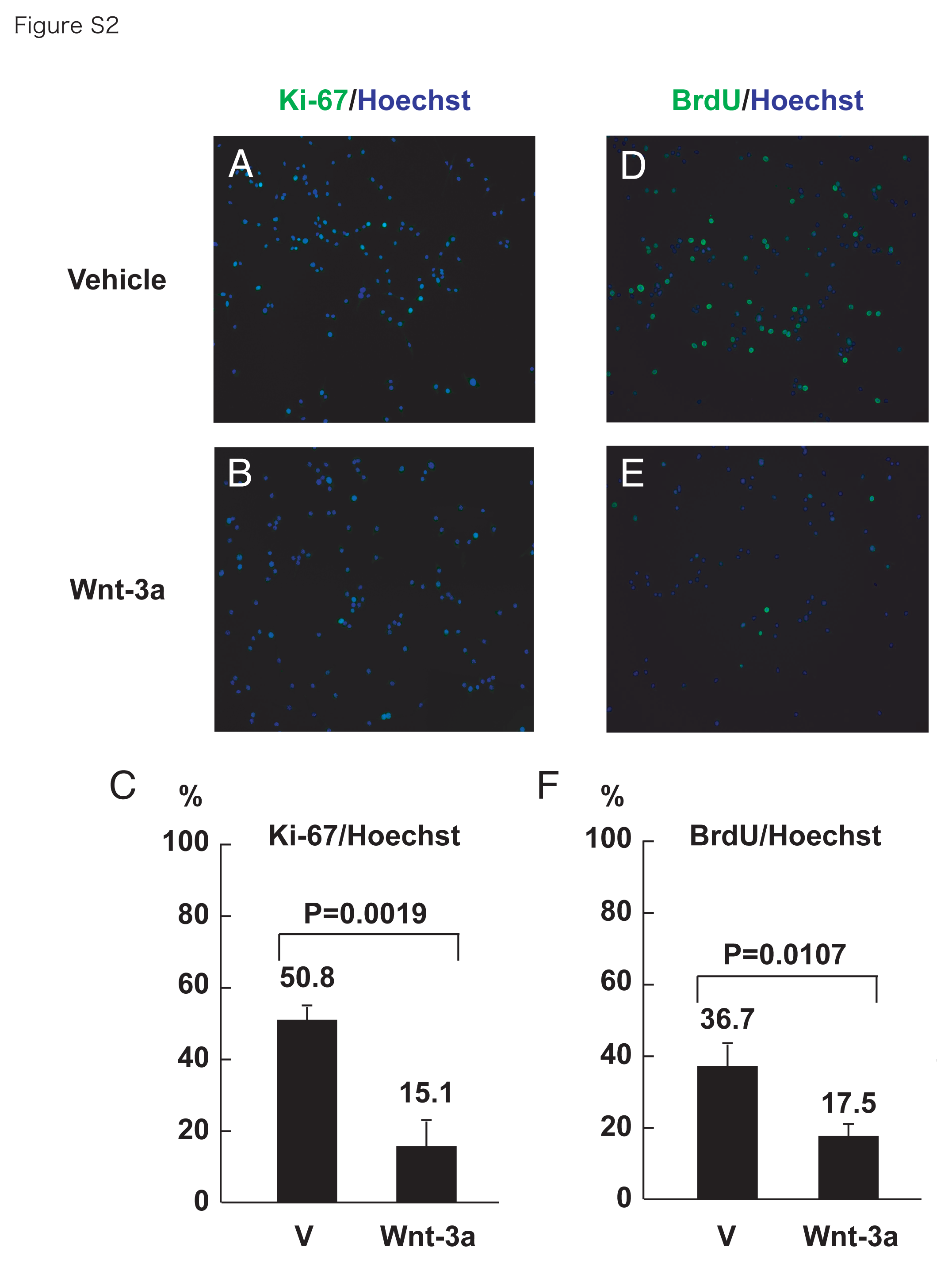

Supplement: Additional file 3: Figure S2 — Wnt-3a inhibits proliferation of adult primary myoblasts. (A-B) Ki-67 antibody staining of primary myoblasts treated with vehicle control (A) and Wnt-3a recombinant protein (50 ng/ml) (B). (C) Percentage of Ki67 positive cells. (D-E) BrdU incorporation of primary myoblast cells treated with control (D) and Wnt-3a protein (E). (F) Percentage of cells incorporated BrdU. [file 2044-5040-3-5-S3.tiff]

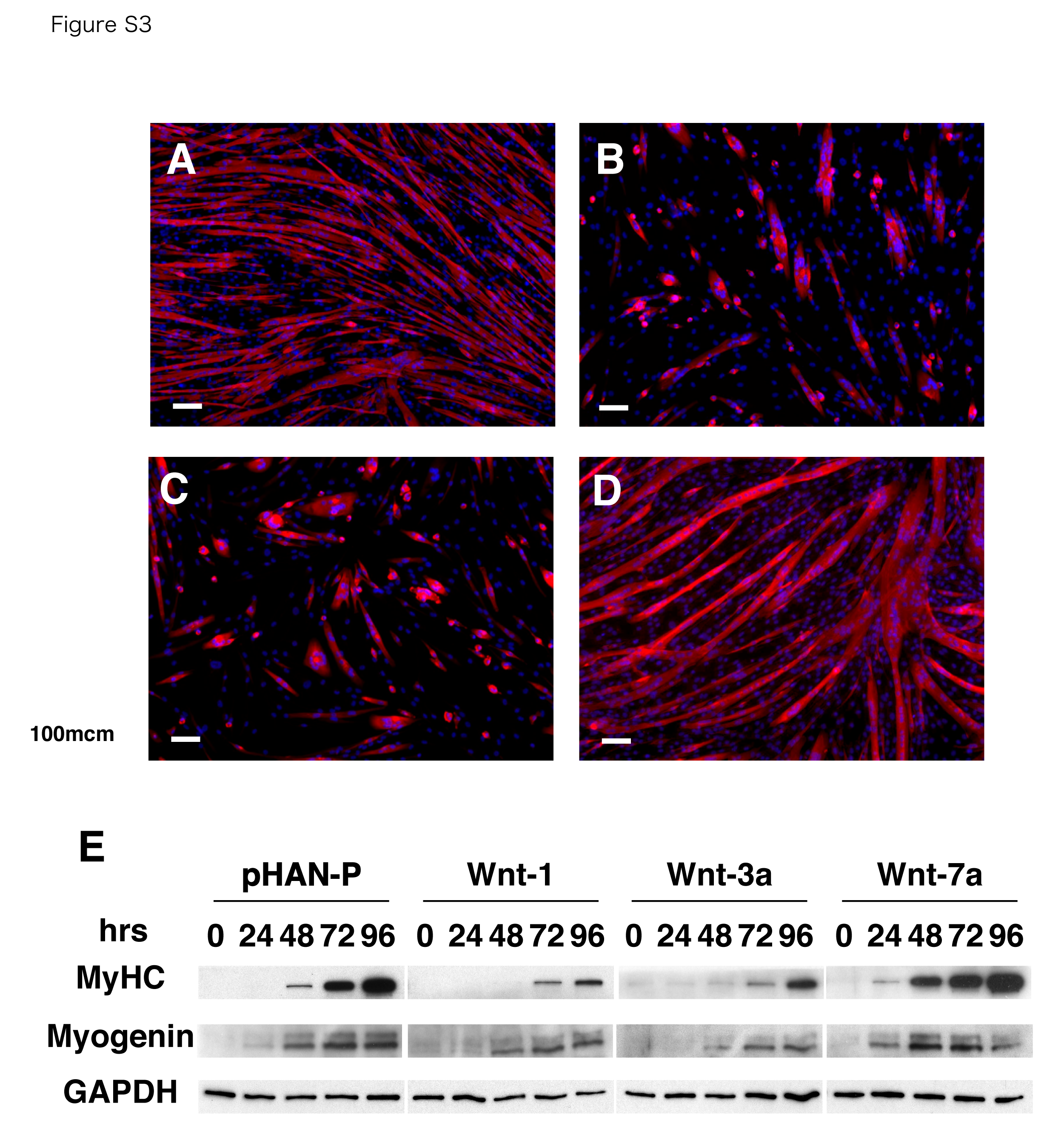

Supplement: Additional file 4: Figure S3 — Overexpression of Wnt-1 and Wnt-3a inhibits the myogenic differentiation of C2C12 myoblasts. (A-D) Immunofluorescence showing sarcomeric myosin heavy chain protein expression in C2C12 cells transduced with retrovirus overexpressing (A) Mock, (B) Wnt-1, (C) Wnt-3a and (D) Wnt-7a after four days of induced myogenic differentiation. (E) Western blotting analysis showing expression of myosin heavy chain (MyHC), myogenin and GAPDH in control, Wnt-1, Wnt-3a and Wnt-7a overexpression C2C12 myoblasts at different time points after induced to differentiate. GAPDH: Glyceraldehyde 3-phosphate dehydrogenase. Scale bar: 100 μm. [file 2044-5040-3-5-S4.tiff]

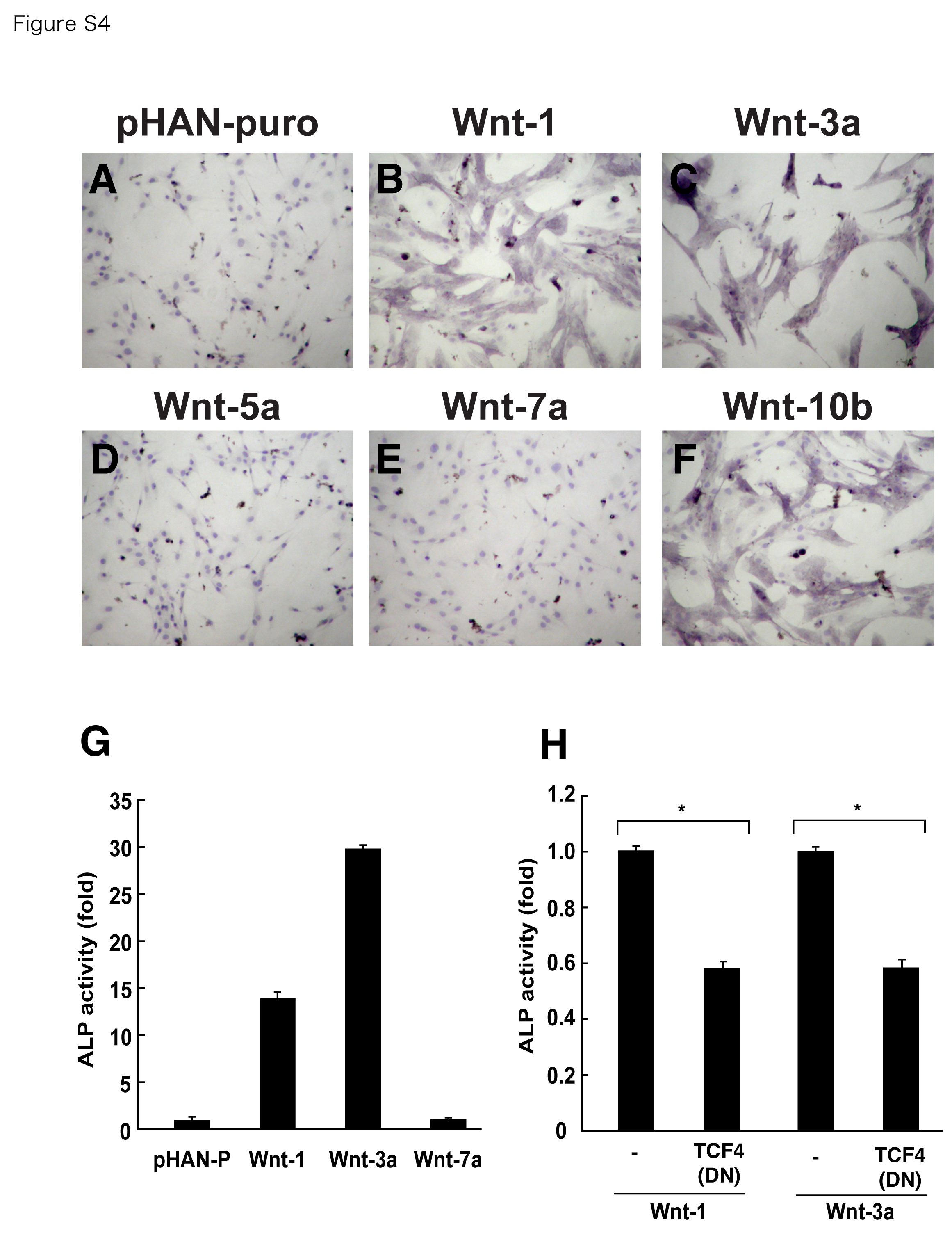

Supplement: Additional file 5: Figure S4 — Canonical Wnts (−1, -3a and 10b) induces alkaline phosphatase (ALP) activity in C2C12 cells. (A-F) ALP staining (Purple signal) of C2C12 cells transduced retrovirus expressing empty vector (Mock) or Wnt vectors as indicated. (G) Relative ALP activity. (H) The dominant negative (DN) TCF-4 suppressed ALP activity in Wnt-1 and Wnt-3a expressing C2C12 cells. [file 2044-5040-3-5-S5.tiff]

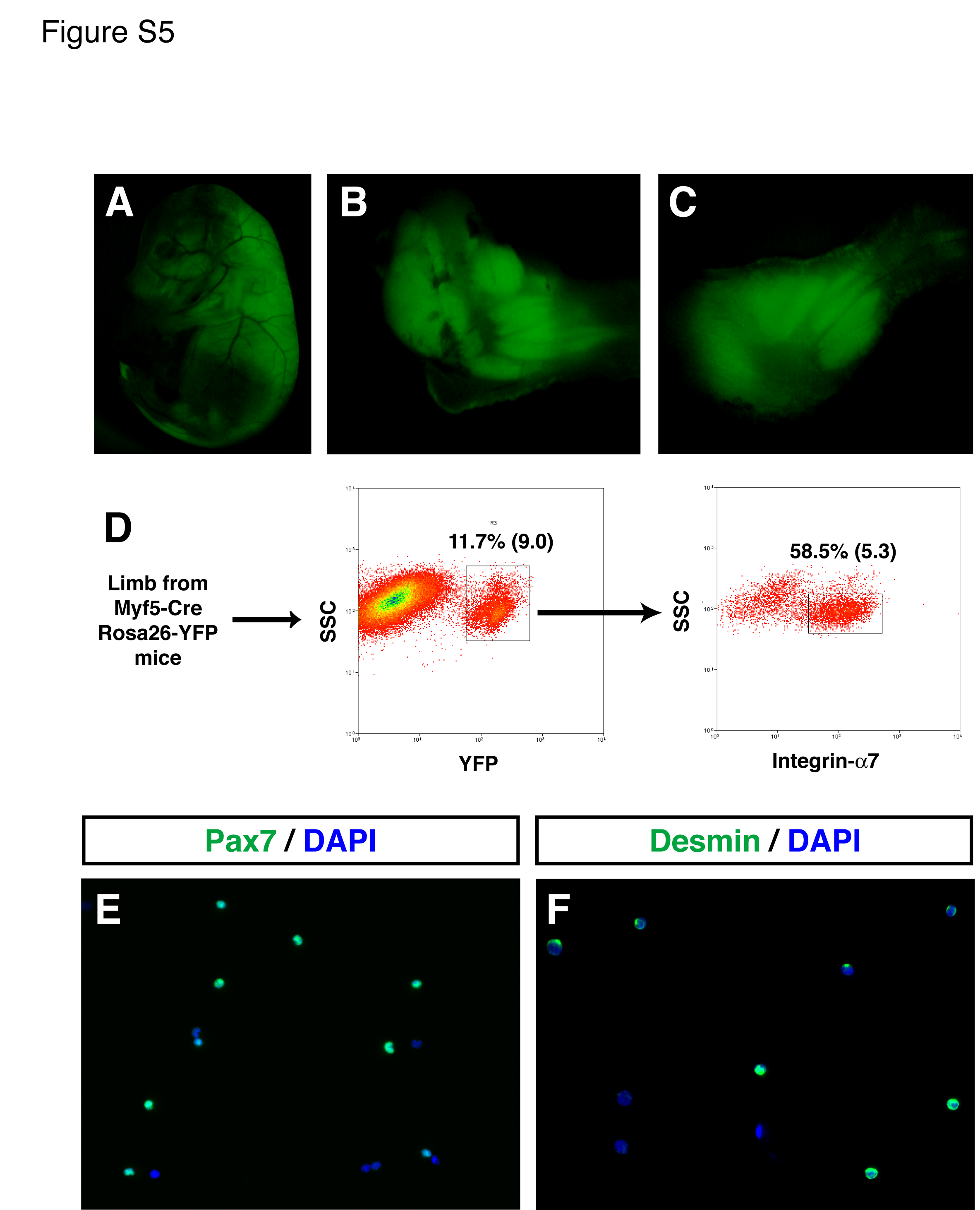

Supplement: Additional file 6: Figure S5 — Isolation of fetal myoblasts from E14.5 embryos of Myf5-Cre/ROSA26-YFP mice by fluorescence activated cell sorting (FACS). (A-C) YFP expression in whole embryo (A), forelimb (B) and hindlimb (C) of E14.5-15.5 Myf5-Cre/ROSA26-YFP embryo. (D) Strategy for isolating fetal myoblasts by FACS. Whole limbs of Myf5-Cre/ROSA26-YFP embryos were minced and digested by collagenase and dispase. Single cells were stained with alpha7-integrin antibody and selected by YFP and alpha7-integrin expression. The sorted YFP and alpha7-integrin double positive cells were stained with antibody for Pax7 and desmin, makers of fetal myoblasts (nuclei were counterstained by DAPI in blue). [file 2044-5040-3-5-S6.tiff]
